# Relationship between self-efficacy and university dropout: a systematic review

**DOI:** 10.3389/fpsyg.2025.1553485

**Published:** 2025-07-30

**Authors:** Ana B. Bernardo, Vanesa García-Gutiérrez, Maria Esteban, Jorge Maluenda-Albornoz

**Affiliations:** ^1^Department of Psychology, University of Oviedo, Oviedo, Spain; ^2^Department of Industrial Engineering, University of Concepción, Concepción, Chile

**Keywords:** self-efficacy, university dropout, higher education, systematic review, protective factors

## Abstract

Self-efficacy is a key determinant of university student persistence, while dropout remains a global challenge. This article is a systematic review of a large body of research examining the relationship between self-efficacy and university dropout. A literature search was started in databases, eligible studies were selected according to inclusion and exclusion criteria and their quality was analyzed, obtaining the 16 documents that were finally analyzed. The results show that the phenomenon of abandonment and the understanding of the different related variables is considered a problem of growing interest in the scientific community. Self-efficacy is a key factor in students continuing their studies. Students with higher self-efficacy are less likely to drop out of university. However, most research in the field is cross-sectional, which limits the understanding of how self-efficacy changes over time. More longitudinal research is suggested to address this limitation and to develop educational policies that strengthen self-efficacy and reduce dropout.

## 1 Introduction

According to United Nations Educational, Scientific and Cultural Organization (UNESCO) reports, the number of students enrolled in universities worldwide has almost doubled in the last 20 years, increasing from 19 to 38% at a global level (UNESCO, 2023). However, the number of students who complete higher education is significantly lower than the number who start university, indicating that many students drop out before completing their studies. The reports indicate dropout rates of around 21%, although, because dropping out of university is a cross-border, persistent phenomenon affecting more than 180 countries around the world. These problem vary by country, with Slovenia, Spain, Italy and Colombia showing the highest dropout rates of 45, 31, 33.2 and 30%, respectively (Ministerio de Universidades, 2022; OECD, 2022).

With the creation of the European Higher Education Area (EHEA), dropout became a quality indicator for universities. This means that many international organizations are currently interested in studying and combatting this phenomenon, including the Organisation for Economic Cooperation and Development (OECD) and UNESCO, who regularly monitor and report the global university situation. Reducing dropout rates and increasing student retention and persistence in the university system has become one of universities’ main concerns (Seligman and Adler, 2018), mainly because of the consequences of dropout. At a social level, it has a notable negative impact on countries’ economic growth (Torrado and Figuera, 2019), human capital development and, consequently, socioeconomic mobility (Morentin-Encina et al., 2019). At institutional level dropout impact as a quality and efficiency indicator (Maluenda-Albornoz et al., 2023). It also has consequences that affect students’ personal and family environments, causing mental health problems—such as anxiety, depression, and low self-esteem—and family conflict (Sosu and Pheunpha, 2019).

Although defining dropout is a complex task, it can be conceptualized as a progressive phenomenon of disengagement from the university system, either permanently or by transfer to another course of study, i.e., by changing course or institution (Almeida et al., 2019; Casanova et al., 2018b).

The first explanatory models of this phenomenon were unidimensional. Their explanation of dropout was based on a single dimension, whether academic, social, or personal cohort (Behr et al., 2020). However, in the 1970s, university dropout began to be considered using multidimensional models, for example, the work of Tinto (1975), a precursor of the interactionist models that explain dropout from a series of academic (performance, engagement, satisfaction, motivation) and personal (previous experiences, student skills, self-efficacy) characteristics that are related to each other and determine the decision to persist or drop out of a course.

This pioneering model has been reformulated by numerous authors, with Bean and Eaton’s (2001) adaptation from a psychological perspective standing out. Those authors argued that the decision to drop out is the result of the interaction between a series of variables of a multicausal nature that arise either from the students themselves or the institutional environment, affected by other academic, social, individual, family or economic factors (Behr et al., 2020; de la Cruz-Campos et al., 2023; Lorenzo-Quiles et al., 2023).

This convergence of factors coincides with the interactionist explanatory models of university dropout (Tinto, 1975; Bean and Eaton, 2001) and with social cognitive theory, according to which, individuals’ behaviors and decisions are the result of a person’s interactions with their environment considering two factors: the individual’s cognition and the external environment they live in (Bandura, 1997; Hamann et al., 2020). One of the notable cognitive factors is the influential role of self-efficacy in regulating behaviors, such that it is a predictive variable of an individual’s professional outcomes, interests, intentions and goals.

Self-efficacy is defined as a person’s beliefs about their ability to complete tasks successfully, which involves goals, behaviors, and environmental conditions (Bandura, 1997; Lin et al., 2018). Within the framework of higher education, self-efficacy comprises five dimensions (Guerreiro-Casanova and Polydoro, 2011): (a) academic self-efficacy—students’ beliefs about their ability and competence to control and execute certain actions that allow them to learn and master tasks and perform at satisfactory levels; (b) self-efficacy in self-regulation—the perception of the ability to establish objectives and goals which promote academic development; (c) self-efficacy in social interactions—referring to students’ perceptions of their ability to establish relationships with peers and teachers and to integrate better academically and socially; (d) self-efficacy in proactive actions—which refers to the perception of the ability to take advantage of opportunities to keep education-related knowledge up-to-date and to undertake actions that promote institutional improvement and, finally, (e) self-efficacy in academic management—referring to confidence in the ability to plan and engage with academic activities in order to complete them.

Academic self-efficacy is related to dropout intention, not only because of its direct relationship with academic performance, but also because it plays a mediating role between dropping out and other cognitive motivational variables, such as engagement, coping strategies, self-regulated learning, satisfaction and academic well-being. In addition to having an impact on behaviors that benefit adaptation to the university context, all these factors ultimately promote academic success (Díaz-Mujica et al., 2022). Along these lines, research indicates that academic self-efficacy is an important predictor of university students’ academic performance (Honicke and Broadbent, 2016). This means that understanding the factors that can influence self-efficacy beliefs is fundamental for designing curricula and teaching methods. Predictor variables for drop out or persistence at university, which have a direct relationship to self-efficacy, include academic performance, a feeling of belonging, academic satisfaction, resilience, well-being, and academic engagement (Bean and Eaton, 2001; Casanova et al., 2018a).

Academic performance is the best predictor variable of dropout (López-Aguilar and Ricardo Álvarez-Pérez, 2021). The better a student’s academic performance, the lower their likelihood of dropping out (Moncada et al., 2019). It is also a mediating variable that bidirectionally influences other affective-motivational variables such as self-efficacy. Research in higher education has confirmed this direct relationship: the greater the feeling of self-efficacy, the better the academic performance and vice versa (Honicke and Broadbent, 2016). In this regard, self-efficacy is a motivational component with positive effects on students’ academic performance; those with a high sense of self-efficacy about their performance in academic tasks exhibit better academic performance (Acosta-Gonzaga and Ramirez-Arellano, 2020).

A student’s feeling of belonging at university is defined as their sense of adjustment and identity with respect to their academic environment (Walton and Cohen, 2007). In this context, the relationship with peers and faculty is a predictor of student persistence at university, since good relationships promote students’ adaptation to the university context and make them more likely to continue their studies (Maluenda-Albornoz et al., 2022). Greater feelings of belonging in the university context—either because of relationships with teachers and peers, or because of support from the institution—has been reported to strengthen levels of self-efficacy, significantly increasing the probability that students continue in higher education and achieve their professional goals (Barrientos-Illanes et al., 2021; Saefudin et al., 2021). This is particularly important for new students (Criollo et al., 2017; Tinto, 1975).

The feeling of belonging and satisfaction are variables that correlate with each other and, in turn, influence academic well-being. In this regard, academic satisfaction is a cognitive-affective evaluation of overall satisfaction with academic experiences (Tomás and Gutiérrez, 2019). According to Self-Determination Theory, students’ satisfaction with their educational environment is broadly related to academic dropout (Deci and Ryan, 2008). High levels of satisfaction are associated with greater effort, performance and achievement on the part of students (Gillet et al., 2019), making students’ satisfaction with their studies a key indicator of their academic success (Suhlmann et al., 2018). Research has highlighted the relationship between student satisfaction and self-efficacy. Students who are satisfied with their academic achievements are more likely to feel effective (Abdous, 2019) and self-efficacy beliefs in turn act as a predictor of success, with a bidirectional relationship between achievement, academic satisfaction and persistence (Lent et al., 2015).

Academic well-being is defined as the individual’s self-perception and self-evaluation of their academic situation and is associated with both the decision to drop out (Năstasă et al., 2022) and with the feeling of self-efficacy. The higher the perception of self-efficacy, the greater the students’ self-esteem and the lower the levels of stress or anxiety toward exams, increasing the likelihood that students will continue their education (Wang et al., 2016). Previous research has shown a direct association between self-efficacy and engagement (Dominguez-Lara et al., 2023), since a high level of self-efficacy is associated with increased student engagement with academic tasks, which in turn is related to academic success (Medrano et al., 2015) and therefore to them continuing with their studies.

Finally, it is worth highlighting the predictive role of resilience—a student’s capacity to successfully face different academic challenges through effective adaptive responses—in students’ decisions to drop out of university courses (Bittmann, 2021; Zumárraga-Espinosa, 2023). Several studies in this field have shown a direct connection with academic self-efficacy; high levels of resilience are associated with greater academic efficacy (Atencia-Oliva et al., 2020; Vizoso-Gómez and Arias-Gundín, 2018). Students with high self-efficacy tend to have high levels of resilience that allow them to better face challenges, make more effort, and persevere in tasks regardless of their difficulty, leading to greater academic success (Metcalf and Wiener, 2018).

As the scientific literature shows, self-efficacy is a predictive factor for university dropout risk, influenced by a multitude of variables—such as academic performance, a feeling of belonging, satisfaction, well-being, and academic commitment—which are in turn correlated with dropout (Bean and Eaton, 2001; Casanova et al., 2018a; López-Aguilar and Ricardo Álvarez-Pérez, 2021). This convergence of variables makes it difficult to establish a relationship between self-efficacy and dropout, so it is important to standardize results to reveal patterns in this relationship.

Due to the impact of self-efficacy on academic success, there have been several systematic reviews in this field. However, they sought to determine the role of self-efficacy in a non-university environment (Patricio-Gamboa et al., 2021), in relation to academic performance (Honicke and Broadbent, 2016), or in a professional environment (Wray et al., 2022). Although Díaz-Mujica et al. (2022) conducted a systematic review looking at the university context, its purpose was to systematize the conceptualization and evaluation of self-efficacy in higher education, but to date, there are no systematic reviews examining the relationship between self-efficacy and university dropout, which is a gap in the knowledge in this scientific field. Considering this, the main aim of the present systematic review is to summarize how the scientific community has analysed the role of student self-efficacy in the decision to drop out of university. The review followed the guidelines of the PRISMA-2020 declaration (Page et al., 2021). In pursuit of the study’s aims, the following research questions were formulated:

1. How many studies published in the last 10 years examined the relationship between university dropout and self-efficacy, where were they done, and with what samples?
2. How are university dropout and self-efficacy conceptualized in these studies?
3. What types of self-efficacy do the selected scientific studies address?
4. How is self-efficacy related to university dropout (moderating, mediating, predictive, etc. relationships)?
5. What measures or recommendations are proposed by the scientific community to improve self-efficacy and prevent dropout?

## 2 Method

This study applied a systematic review methodology to explore and summarize research findings on the relationship between self-efficacy and university dropout. The systematic review consisted of several phases: literature search; selection of eligible studies according to inclusion and exclusion criteria; analysis of study quality; and coding of studies.

### 2.1 Search strategy and eligibility criteria

The literature search was carried out in 2024 using the Web of Science and Scopus databases. The searched gareded scientific studies with focus on the relationship between university dropout and self-efficacy. To do this, several search terms were combined in both Spanish and English using Boolean operators as follows: (“self-efficacy” OR “self efficacy” OR “selfefficacy” OR “autoefficacy” OR “autoeficacia”) AND (“retention” OR “drop out” OR “dropout” OR “permanence” OR “retención” OR “abandono” OR “permanencia”) AND (“University” OR “Higher education” OR “University” OR “Universi*” OR “educación superior” OR “educación terciaria”).

The systematic review focused on the results that met the following inclusion criteria: (a) undergraduate students in any study program and any kind of university; (b) empirical studies on university dropout/retention and self-efficacy written in English, Spanish or Portuguese and published between 2014 and 2024; (c) articles relating university dropout/retention and self-efficacy; (d) studies using quantitative or mixed methodologies. The following exclusion criteria were applied: (a) studies with online students; (b) articles only looking at university dropout or at self-efficacy.

### 2.2 Quality criteria

The quality criteria were established following the parameters described by Camacho et al. (2021). Seven indicators were set addressing the research justification of the study, the theoretical basis, quality, reliability, validity, and consistency and adequacy of the findings (see Table 1).

**Table 1:** Methodological quality indicators (MQIs).

Standard	Quality criteria
Provides a clear argument that links theory and research and demonstrates a coherent chain of reasoning. Explains theoretical and previous research in a way that supports the formulation of the questions.	(1) Explain theory and/or previous research in a manner that informs the formulation of purposes/objectives that can be explored empirically.
	(2) Establish explicit links between the results and previous theory and research or argue in favour of the study.
It uses a rigorous, systematic, objective methodology to produce reliable, valid knowledge relevant to educational activities and programs.	(3) Ensure that methods are described in sufficient detail and clarity so that procedures can be clearly visualized. Data collection should be described in such a way that readers can replicate the procedures in a quantitative study or trace the data analysis in a qualitative study.
	(4) Provide evidence of reliability. Has this evidence been provided for the data collected? Has the investigator provided information on the development of the development and study populations?
	(5) Provide evidence of validity: Has this evidence been provided for the data collected? Is there information on the development and adaptation of the instrument for specialized populations?
	(6) Description of participants Was the sample well characterized?
Presents findings and makes assertions that are appropriate and supported by the methods used.	(7) The results and conclusions are legitimate or consistent with the data collected.

### 2.3 Data extration

This systematic review made a qualitative summary of quantitative studies. To that end, a worksheet was designed to systematically extract the most important data from the selected studies. The table contained the following categories: (1) key words; (2) sample characteristics; (3) conceptualization of university dropout and self-efficacy; (4) study design; (5) analysis of the relationship between dropout and self-efficacy; and (6) proposed measures to improve self-efficacy and reduce university dropout.

Data from the all articles included in the review were extracted by one of the researchers and validated by another. During the process, the information was coded in a worksheet that collected the key words indicating the main themes of each article in order to identify which terms were most frequently used. With respect to the sample, the country and the number of participants were considered. For the conceptualization of university dropout and self-efficacy, we retrieved the definitions offered by the articles for these terms, indicating only the definitions from articles with an explicit definition, thus avoiding inferring the conceptualization of these concepts through definitions implicit in the text. We also collected the types of self-efficacy examined by the authors and coded the research design, considering whether it was longitudinal or cross-sectional. For each study, we recorded the variables studied that were related either to self-efficacy or to dropout, and the results on the relationship between self-efficacy and dropping out of university or, where applicable, the intention to drop out. Finally, the measures that authors proposed to improve self-efficacy and prevent dropout in higher education were recorded.

### 2.4 Bias assessment

The PRISMA protocol (Page et al., 2021) was used throughout the process of analysing the selected studies, with particular attention given to assessing and minimizing the risk of bias. Therefore, several researchers with expertise in the field of university dropout reviewed both the keywords and the inclusion and exclusion criteria. Similarly, the study identification process was carried out independently by two researchers, reaching full consensus on the studies to be included. Finally, the quality assessment and data extraction processes were validated by two researchers.

## 3 Results

The aim of this review was to determine the relationship between self-efficacy and the intention to drop out of university courses. To select the studies, several criteria were used to narrow the search, seeking only open-access scientific articles published between 2014 and 2024 in Spanish, English or Portuguese. The search yielded a total of 2,654 records. Subsequently, initial screening was performed based on the titles, eliminating studies examining other educational stages, studies about teachers´ self-efficacy, self-efficacy in the workplace, and studies that were not related to university dropout. After analysing the titles, 122 articles were selected; duplicate studies were eliminated. The final data set comprised 104 records.

Then, the inclusion and exclusion criteria were applied by two researchers who assessed the abstracts of the 104 initially selected studies, excluding a total of 56 studies. The remaining 45 studies were then analysed in depth to verify that they met the eligibility criteria, so that the final data set included 16 articles.

**Figure 1:**
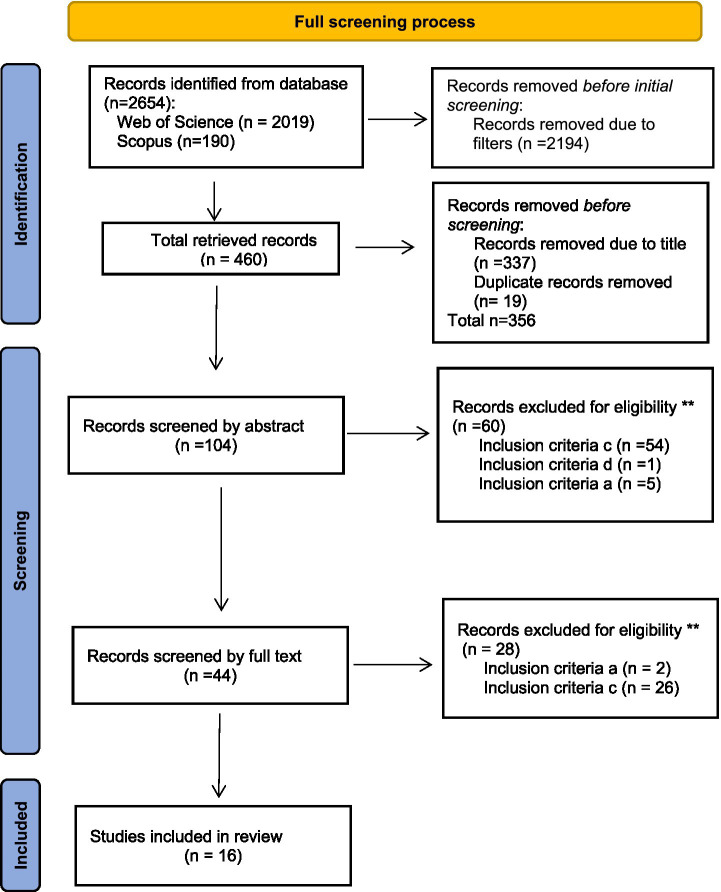
Systematic review selection process.

Figure 1 shows the PRISMA diagram, which is an overview of the search process used.

Two experts assessed the quality of each of the 16 selected studies using the template in Table 1 (Miller et al., 2016). For each study, the experts awarded 1 point if a quality criterion was met and 0 points if the criterion was not met or could not be confirmed. The final quality score was the sum of the points, with high quality if the total score was 6 points or more (Camacho et al., 2021). The lead author evaluated 100% of the studies and the second author evaluated 30% at random, reaching a concordance between both evaluators of 100% for all the quality criteria (see Table 2). This concordance may be because the articles chosen were published in high-impact journals and had therefore already passed the relevant quality filters, such as a double-blind peer review.

**Table 2:** Quality of the articles included in the review.

Study	(1) Explaining theory	(2) Links results-theory	(3) Methods	(4) Reliability	(5) Validity	(6) Participants	(7) Results/conclusions	Total score
Barrientos-Illanes et al. (2021)	1	1	1	1	1	1	1	7
Buizza et al. (2024)	1	1	1	1	1	1	1	7
Cădariu and Rad (2023)	1	1	1	1	1	1	1	7
Girelli et al. (2018)	1	1	1	1	1	1	1	7
Gonzalez-Perez et al. (2022)	1	1	1	1	1	1	1	7
Heritage et al. (2023)	1	1	1	1	1	1	1	7
López-Aguilar and Ricardo Álvarez-Pérez (2021)	1	1	1	1	1	1	1	7
López-Angulo et al. (2022)	1	1	1	1	1	1	1	7
Marczuk and Strauss (2023)	1	1	1	1	0	1	1	6
Morelli et al. (2021)	1	1	1	1	1	1	1	7
Morelli et al. (2023)	1	1	1	1	1	1	1	7
Nemtcan et al. (2020)	1	1	1	1	1	1	1	7
Nemtcan et al. (2022)	1	1	1	1	1	1	1	7
Óturai et al. (2023)	1	1	1	1	1	1	1	7
Van Herpen et al. (2017)	1	1	1	1	1	1	1	7
Vidal et al. (2022)	1	1	1	1	1	1	1	7

The results are organized according to the research questions. Supplementary Table 1 shows the worksheet on which the data extraction was performed.

### 3.1 How many studies published since 2014 are related to both university dropout and self-efficacy, where were they done, and with what samples?

On examining the Web of Science and Scopus databases, there were a total of 460 articles published in open access in the last decade. However, after applying the inclusion and exclusion criteria, this number dropped to 45, although in the end only 16 had examined the relationship between dropout and self-efficacy in undergraduate students and had sufficient methodological quality to be analysed.

**Figure 2:**
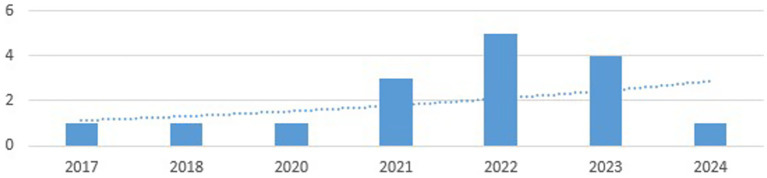
The number of published articles about dropout and self-efficacy (2017–2024).

It was observed that the scientific production in this field is increasing, with a greater proliferation of research in this field observed in the last 4 years, with the largest volume of studies concentrated in 2022 (see Figure 2). However, it should be noted that the systematic review was carried out in the first half of 2024, so the data for that year may not be representative.

These studies were carried out at universities in various countries, including: Italy (4), Norway (3), Chile and Spain (2), Romania, Australia, Germany, the Netherlands and Ecuador (1).

Concerning sample size, 6.25% of the studies used samples of between 0 and 100 participants, and the same percentage used between 300 and 400 students, 800 and 900 students, between 900 and 1,000 students, and more than 1,000 students. 12.50% of the studies were conducted with a sample of between 100 and 200 participants, and the same percentage of studies used samples in the ranges 200–300, 600–700, and 700–800 participants. Finally, 25% of the studies were done with samples of between 400 and 500 students.

### 3.2 How are university dropout and self-efficacy conceptualized?

Seven of the 16 analysed articles did not offer a conceptualization of dropout. Two studies resorted to terms such as retention or persistence. In these cases, Barrientos-Illanes et al. (2021) defined the intention to drop out together with the intention to persist and conceptualized it as the student’s assessment of the possibility of discontinuing or continuing with their university course. Heritage et al. (2023), used the term retention as a synonym for other related terms, such as student withdrawal, desertion and dropout, understanding retention as the situation where a student stays at university until they complete their education and gain a university qualification.

Two of the studies distinguished between dropping out (leaving the educational system completely) and transferring (moving from the university where students began their course to a different higher education institution).

Considering the definitions from the other articles, dropout could be conceptualized as a general construct that refers to a dynamic, cumulative and multifactorial student process of progressive disengagement that develops in the early stages of the university experience where students make a series of decisions about whether or not to continue with their course of study. Dropping out is not a single event, but rather the final phase of a dynamic process involving the convergence of multiple factors (lack of motivation, thoughts, desires and intentions) that students experience about the possibility of withdrawing from higher education before graduation, without concrete intentions of returning.

In the case of self-efficacy, only one study did not include the conceptualization of this term. The remaining 15 studies used Social Cognitive Theory, proposed by Bandura (1997), to define self-efficacy as the set of beliefs, perceptions, or expectations students have about their own abilities and skills to achieve their goals and successfully complete their tasks in the academic setting. Thus, academic self-efficacy is understood as the individuals’ judgments about their ability to effectively organize and execute the necessary actions to achieve academic success. This implies having the ability, self-confidence and perceived capacity to grasp and predict social situations, expressed through behavior that is demonstrative of the individual’s adaptation to social situations.

### 3.3 What types of self-efficacy do the selected scientific studies address? What kind of design do these studies follow?

In terms of the kind of self-efficacy addressed by the research, 12 studies focused on analysing the relationship between dropout and academic self-efficacy, while 3 opted for self-efficacy in self-regulation. Only 1 study analysed social self-efficacy and its relationship with dropout.

Looking at research design, one study was a systematic review, while the rest of the studies were analysed according to research design. The vast majority (15 studies) opted for a cross-sectional study design and only 1 was longitudinal.

### 3.4 How is self-efficacy related to university dropout?

The effects of self-efficacy on dropout were analysed based on the results of the studies, paying special attention to its mediating, moderating, or predictive role. Self-efficacy had a clear relationship with dropout and was a mediating variable in 9 of the 16 studies. In these cases, self-efficacy mediated between dropout and other variables such as satisfaction, perceived autonomy support, self-regulated knowledge, intrinsic motivation, academic adjustment, performance, engagement, procrastination and previous academic achievement.

Academic self-efficacy showed a significant positive correlation with students’ satisfaction with their courses, i.e., participants with higher levels of academic self-efficacy tended to experience greater satisfaction with their courses of study and their intention to continue their education was greater.

In contrast, controlling for the effects of learning strategies and intrinsic motivation, self-efficacy was inversely related to university dropout. For example, first-year students with good sense of self-efficacy were less likely to dropout, and making use of learning strategies such as self-regulation, concentration, active support, time management, effort, was also related with lower dropout.

Both autonomous motivation and self-efficacy were significantly and negatively associated with the intention to drop out. In addition, self-efficacy mediated the effects of perceived parental and teacher support for autonomy on dropout intention, such that students who attended university for more autonomous reasons and had stronger beliefs in their academic abilities were less likely to develop intentions to dropout.

Self-efficacy acted as a mediator in the relationship between the intention to persist at university and variables such as engagement, performance, and academic adjustment. In these cases, there were positive relationships between these variables and self-efficacy and an inverse relationship with university dropout, i.e., students who had a higher degree of academic engagement, performance or adjustment and who, in turn, experienced higher levels of self-efficacy, were less likely to drop out of higher education. However, academic self-efficacy was found to be negatively related to procrastination and dropout intentions, i.e., students’ academic self-efficacy was significantly related to dropout intentions through academic procrastination.

Four studies showed a moderating role of self-efficacy in the negative relationship between dropout and variables such as engagement and performance, well-being, self-regulation, adaptive capacity, and satisfaction.

Student well-being was related to self-regulation of learning and self-efficacy. High levels of self-efficacy decrease likelihood of student dropout and fostered both student adaptation and a coping attitude, enhancing engagement to cope with challenges, emotionally stressful situations and burnout, thus reducing the risk of dropout.

Looking at satisfaction, studies showed that students with high levels of social self-efficacy maintained relationships or social contacts more easily, which suggests a better degree of socialization and feeling of belonging leading to an increased desire to stay at university.

One study showed that self-efficacy played a mediating and moderating role between locus of control, engagement, academic interaction, and satisfaction. The results showed that the intention to drop out of university was negatively related to self-efficacy, institutional commitment, and academic integration, and positively related to an external locus of control. In other words, intention to drop out of university was related to low self-efficacy for self-regulated learning, low subsequent institutional engagement, poor academic integration with other students, and higher external locus of control. On the other hand, dissatisfaction with the university experience was associated with lower self-efficacy for self-regulated learning, lower subsequent institutional commitment and poorer academic integration with other students.

Finally, 2 studies supported the existence of a direct relationship between self-efficacy and dropout. Overall self-efficacy coefficients were a significant predictor of student dropout. However, both studies indicated that this relationship was stronger when considering a series of additional variables that influence academic performance. For example, factors such as academic grades and overall student performance, as well as psychological and emotional elements such as intrinsic motivation, test anxiety, and attributions of high performance, were presented as mediating variables that enhanced the relationship between self-efficacy and dropout. Hence, self-efficacy was not only a direct predictor, but also a variable whose effect was reinforced by the presence of these factors, suggesting that the indirect relationship between self-efficacy and dropout may be even more significant when multiple dimensions of the academic and personal context are considered.

### 3.5 What measures or recommendations do the scientific community propose to improve self-efficacy and prevent dropout?

All the researchers agreed that dropping out of university is a complex problem that must be prevented due to its negative consequences. To that end, we compiled the measures or recommendations for increasing university students’ self-efficacy and for contributing to the design and implementation of actions to prevent the phenomenon of dropping out.

Twelve of the 16 studies provided some proposals or recommendations that, at the institutional or classroom level, could increase levels of student self-efficacy and therefore contribute to reducing dropout or increasing rates of student persistence.

The proposed measures are grouped into 4 large blocks (see Table 3) that include: measures targeting family members (1); interventions with university students (2); measures targeting teachers (3); and measures for educational institutions and policies (4).

**Table 3:** Recommendations for improving self-efficacy and preventing dropout.

Block	Measures
Measures targeting family members	Promotion of training programs that encourage autonomy-supporting behaviors in parents (Girelli et al., 2018). Involve families in the educational process (Kocsis and Molnár, 2024).
Interventions with university students	Accompaniment at different levels (Buizza et al., 2024). Improve students’ ability to regulate themselves, focus on academic support, new study methods, and better self-organization (Buizza et al., 2024; Kocsis and Molnár, 2024; López-Aguilar and Ricardo Álvarez-Pérez, 2021). Psychological counseling to cover social, psychological, and educational support needs (Buizza et al., 2024; Kocsis and Molnár, 2024; Lorenzo-Quiles et al., 2023). Professional advice (Heritage et al., 2023). Provide targeted interventions and support programs to promote academic persistence and success (Cădariu and Rad, 2023). Create an environment that fosters satisfaction and commitment to the chosen field of study (Cădariu and Rad, 2023; Morelli et al., 2021, 2023). Promote autonomous motivation and perceived self-efficacy (Girelli et al., 2018; Óturai et al., 2023; Morelli et al., 2021, 2023; Vidal et al., 2022). Support students’ autonomy in the educational context (Girelli et al., 2018). Tutoring to improve academic skills (Kocsis and Molnár, 2024; Marczuk and Strauss, 2023; Nemtcan et al., 2022). Foster a supportive environment among peers (Kocsis and Molnár, 2024; Morelli et al., 2021, 2023). Provide regular and constructive feedback (Kocsis and Molnár, 2024). Programs that foster personal and emotional development (Gonzalez-Perez et al., 2022; Kocsis and Molnár, 2024).
Measures targeting teachers	Encourage teacher behaviors that improve student autonomy and facilitate their academic adjustment (Girelli et al., 2018). Conducting classroom activities in which success is recognized (Gonzalez-Perez et al., 2022). Encourage faculty to focus on student success and their ability to perform challenging tasks (Gonzalez-Perez et al., 2022). Provide students with authentic assessment and work-integrated learning opportunities (Heritage et al., 2023). Provide precise indications on the study material and objectives of the course (Morelli et al., 2021, 2023). Periodic feedback on study and learning strategies (Kocsis and Molnár, 2024). Attribution retraining programs (Morelli et al., 2021, 2023; Vidal et al., 2022).
Measures for educational institutions and policies	Implement actions and programs at the university level to improve self-efficacy and academic motivation (Girelli et al., 2018; Kocsis and Molnár, 2024). Interventions to foster a growth mindset through positive emotions (Cădariu and Rad, 2023; Gonzalez-Perez et al., 2022; Girelli et al., 2018). Develop positive psychological programs to influence students’ self-evaluation criteria (Gonzalez-Perez et al., 2022). Provide realistic previews of courses for informed decisions (Heritage et al., 2023). Offer university orientation weeks (Heritage et al., 2023). Encourage student groups and associations (Heritage et al., 2023). Tutoring and Mentoring Programs (Gonzalez-Perez et al., 2022; Kocsis and Molnár, 2024). Minimize inequalities and provide support measures for disadvantaged groups (Lorenzo-Quiles et al., 2023; Marczuk and Strauss, 2023; Vidal et al., 2022). Improve the quality of teaching, curriculum design and the provision of resources adapted to student needs (Cădariu and Rad, 2023; Vidal et al., 2022). Implement preventive and intervention programs to reduce academic failure and promote well-being (Buizza et al., 2024; Morelli et al., 2021, 2023). Academic tutoring programs focused on academic skills (Kocsis and Molnár, 2024; Nemtcan et al., 2022).

As Table 3 shows, involving the family in the educational process could promote students’ autonomy and academic well-being. This involvement could be through training programs that give families tools to recognize and validate their children’s feelings and perspectives and offer them resources and information to encourage autonomous decision-making, reinforcing student motivation and confidence. In addition, family collaboration with educational institutions could be beneficial in addressing challenges related to student stress, anxiety, or lack of motivation.

The measures aimed at university students included the proposal of interventions to promote social and academic integration, such as mentoring and tutoring programs, study groups, and student associations that promote interpersonal relationships and a sense of belonging, and in turn contribute to fostering academic autonomy and persistence.

The studies demonstrated particular interest in providing psychological interventions and programs addressing aspects such as stress management, anxiety, and academic burnout, intending to improve emotional well-being. They also seemed to value the idea of counseling students at the academic level—such as teaching study skills, time management and problem solving—and at the professional level by offering practical learning opportunities—such as internships—to connect academic content with students’ professional aspirations.

The recommendations highlighted teachers’ roles in the development of students’ self-efficacy and autonomy. The suggestions included implementing teacher training protocols that enable teachers to adapt to the individual needs of students and be able to create a learning environment with practices that promote students’ acquisition of competencies. The studies’ recommendations included, among other measures, training workshops for teachers where they learn to incorporate fundamental techniques for reinforcing student self-efficacy (such as promoting student autonomy, recognizing signs of emotional exhaustion, and applying appropriate support strategies), motivating students, offering students continuous and constructive feedback on academic performance, and providing learning strategies that help students understand that academic skills can be improved to achieve success and complete their education.

Finally, from an institutional perspective, it is essential to develop educational policies that create an academic environment conducive to student satisfaction and engagement. This involves improving the quality of teaching and curriculum design, ensuring that resources and services are tailored to students’ needs and interests. In addition, educational policies should promote specific interventions aimed at reducing academic failure and fostering student well-being while minimizing potential resource inequalities among students, especially in contexts where the technology gap remains an obstacle. There were proposals for interventions that include support programs tailored to the characteristics of each student; i.e. preventive programs that address academic performance problems, and learning opportunities that promote persistence through the recognition of student achievements. Finally, the studies emphasized the need for educational institutions to collaborate to facilitate students’ transition to the academic environment, for example, through programs and services that support students’ academic, personal and professional development, to ensure their long-term success, or by providing clear and realistic information about the courses offered, as well as university orientation weeks.

## 4 Discussion

The present systematic review was prompted by the lack of scientific production examining the relationship between university dropout and self-efficacy. This study aimed to determine the role of self-efficacy in the complex phenomenon of university dropout. To achieve this, an exhaustive search of the literature related to this field was undertaken and 16 articles addressing the topic were analysed.

The review sought to answer a series of research questions. In response to the first question, we found that even though scientific production in the field of dropout is very prolific, few studies have analysed the relationship between dropout and self-efficacy and the bulk of research over the last decade has been in the last 5 years. This suggests that studies in this field are of increasing interest to researchers. Previous reviews with a similar focus of interest have also noted this trend (de la Cruz-Campos et al., 2023; Lorenzo-Quiles et al., 2023), hence the phenomenon of dropout—and understanding the different related variables—is a problem of growing interest in the scientific community. However, the review found that most of the studies were cross-sectional, and there have been hardly any longitudinal studies providing information on how the variables involved in the decision to drop out change over time.

Our second question aimed to determine how self-efficacy and dropout were conceptualized, using the definitions provided by the authors of the studies. We found that the vast majority of studies defined self-efficacy following the Social Cognitive Theory definition proposed by Bandura (1997). This finding is especially relevant, since the convergence in the definition of the construct allows for the establishment of a common and coherent theoretical basis in this area, which favors the evaluations carried out referring to the same concept. However, a wider variety of definitions emerged, although all of them agreed in conceptualizing dropout as a gradual, progressive phenomenon, which happens at different times and spaces where several factors interact (Casanova et al., 2018b). In addition, some authors differentiated between dropout (definitive disengagement) and transfer (change to another degree or university), considering dropout as a more specific concept depending on the student’s decision.

The lack of a clear consensus in the conceptualization of withdrawal university studies represents an important limitation, as it makes it difficult to identify the phenomenon, understand its causes or design effective interventions to prevent it- Likewise, the lack of conceptual heterogeneity hinders the comparison of studies and the generalization of the results obtained in different investigations. A rigorous and coherent operationalization of both constructs could be key to strengthening the validity of studies in this area and thus be able to design evidence-based interventions ensuring the universality of measures and results. This aspect is especially important in the case of dropout, where it is suggested to differentiate between dropout and transfer and to incorporate an administrative measure that allows early identification of students at risk of dropping out. The adoption of these measures would contribute to a more precise conceptualization of the dropout phenomenon, allowing the development of interventions that are better adjusted to the students’ needs. Likewise, reaching a consensus on the conceptualization of both constructs would facilitate future meta-analyses that would help to consolidate a more robust theoretical basis and to support the design of interventions backed by scientific evidence.

The third research question was about determining the types of self-efficacy that have been the object of study in this field. As expected, most of research we analysed was aimed at studying academic self-efficacy and, to a lesser extent, self-regulatory and social self-efficacy. These results make sense, since the relationship of this variable with dropout is studied from a psychoeducational perspective, and previous studies have shown academic self-efficacy to be a predictor of dropout intention (Cădariu and Rad, 2023).

The fourth research question aimed to determine how self-efficacy is related to university dropout. The review showed a direct relationship between self-efficacy and dropout, although there are many variables that affect the decision to drop out (Fortes et al., 2022; Tinto, 2017). Self-efficacy tends to behave as a mediator of other motivational cognitive variables, such as satisfaction, performance, well-being, engagement, autonomy, and self-regulation, which in turn have a positive impact on the individual and promote adaptation to the university context, all factors that ultimately promote academic success (Díaz-Mujica et al., 2022). These results are in line with previous studies confirming a direct, negative relationship between dropout and self-efficacy, i.e., the greater the feeling of self-efficacy, the lower the likelihood of dropout (Cordova et al., 2014; Girelli et al., 2018). Studies agree in highlighting the existence of this relationship, however, these findings contrast with other studies reporting that high self-efficacy has detrimental effects on student performance, since high levels of self-efficacy decrease the degree of effort, which in turn negatively affects performance (Vancouver and Kendall, 2006; Vancouver et al., 2002).

Finally, the last question sought to determine what measures or recommendations the scientific community has proposed for improving self-efficacy and preventing dropout. Of the studies examined, 82.35% proposed measures for increasing higher-education students’ levels of self-efficacy and preventing dropout. These proposals were grouped into 4 blocks according to who they were aimed at: students, families, teachers and educational institutions or policies (see Supplementary Table 1). It is clear that addressing self-efficacy requires a multifaceted approach and the collaboration of the entire educational community to ensure students’ well-being and satisfaction (Buizza et al., 2024; Kocsis and Molnár, 2024), to foster their overall academic adjustment, and to reducethe likelihood of them dropping out (Cădariu and Rad, 2023).

The findings of this systematic review offer a global picture of the state of the art and reaffirm the importance of situating self-efficacy as a keystone of study due to its relationship, not only with dropout, but also with other variables involved in the phenomenon. In recent years this topic has gained interest, however, the small amount of research in this field makes it difficult to establish an explanatory model for the role of self-efficacy, a line of research that could be of interest for the scientific community. The review shows that the conceptualization of self-efficacy shows greater consistency in the scientific field, based mainly on Bandura’s Social Cognitive Theory (1997), which favors the accumulation of knowledge. In this line, academic self-efficacy is the most studied type of self-efficacy, being identified as a protective factor against dropout. On the other hand, dropout presents more heterogeneous definitions, which is a barrier to the comparison of results and the design of effective prevention interventions. The review confirms the relationship between dropout and self-efficacy, highlighting the importance of enhancing university students’ levels of self-efficacy to reduce dropout rates.

As a final conclusion, the studies conducted in the last 10 years on the role of self-efficacy in dropout show that it has a positive impact on dropout prevention. However, although the review highlights the need to recognize and enhance the positive influence of self-efficacy, the lack of specific studies on this variable could be seen as one of the limitations of the review, since that makes it impossible to achieve a complete, objective picture of how self-efficacy independently affects students’ intentions to disengage from the university system.

Another possible limitation of the study is related to the search for studies, since the search terms used could have restricted the number of studies likely to be of interest for the review. In addition, despite efforts to minimize bias in the selection and extraction of data, the heterogeneity of the results may also be considered a limitation, since grouping the diversity of data offered into categories of subjects may have generated errors when summarizing and generalizing the results, which could affect the validity of the results of the review.

While interpreting the findings of this review, it is important to consider the potential presence of biases in the field of research. One of the most important biases is publication bias, since it is possible that the studies that did not find a relationship between self-efficacy and dropout could be underrepresented in the scientific literature and were not available in the databases consulted. This possible bias is considered a limitation in our review and could have generated an overestimation of the effects of self-efficacy on dropout. Therefore, it is necessary for future research to specify its transparency criteria to minimize this type of bias. One suggested line of research for the future is to continue investigating the potential of self-efficacy as a protective agent against dropout, since research in this field is still limited, especially in the case of students who have moved beyond their first year. In view of the results, it would also be advisable to perform more longitudinal studies to check how students’ feelings of self-efficacy vary throughout their university experiences, since most of the studies in the review were cross-sectional. The absence of longitudinal studies constrains the possibility of establishing causal relationships and understanding the evolution of the dropout phenomenon and its relationship with self-efficacy. It is therefore interesting to encourage the development of longitudinal studies that incorporate multiple measurements to identify the predictors of dropout with greater precision and to identify the trajectory of students from the time they enter until they complete their studies. In this sense, future studies could apply experience sampling to offer real-time data on the motivational state of students, providing a better understanding of the dynamics of self-efficacy and the process of academic disengagement. In turn, it would be interesting to combine longitudinal studies with cross-sectional analyses that examine how self-efficacy may predict dropout, and vice versa.

Finally, it would be interesting to conduct future studies that focus on the analysis of new variables that may interfere with the relationship between self-efficacy and dropout, such as socioeconomic status, institutional support, and mental health. The inclusion of these factors would allow a more precise contextualization of this relationship as well as the identification of possible mediating or moderating variables that would help to better understand the conditions under which self-efficacy influences dropout.

## Data Availability

The original contributions presented in the study are included in the article/Supplementary material, further inquiries can be directed to the corresponding author.
