# Supplementary material for: Relationship between self-efficacy and university dropout: a systematic review
Source: Front Psychol. 2025 Jul 30;16:1553485. doi: 10.3389/fpsyg.2025.1553485 (PMC12345370; doi:10.3389/fpsyg.2025.1553485)
Supplement: Supplementary file 1 [file Table_1.docx]

Table S1. *Data extraction*

| Study | Sample and country | Conceptualization | | Self-efficacy type and operationalization | Design | Dropout-to-self-efficacy ratio | Variables influencing self-efficacy | Dropout/self-efficacy results | Proposed measures |
| --- | --- | --- | --- | --- | --- | --- | --- | --- | --- |
|  |  | Intention to drop out/college dropout | Self-efficacy |  |  |  |  |  |  |
| Barrientos-Illanes et al. et al., 2021 | Chile  273 | Intention to drop out: possibility that a student evaluates with respect to interrupting university studies. | Students' beliefs about their own abilities to perform in the academic environment. That is, it refers to the judgments about the ability to organize and execute  the actions necessary for academic success | Academic | Transversal | Mediator | - Perception of autonomy support - Academic satisfaction | Academic satisfaction mediates the relationship of perceived academic self-efficacy on intention to stay in school | None |
| Buizza et al., 2024 | Italy  790 | Not defined | Belief in one's ability to perform a task successfully | Academic | Transversal | Mediator | Self-regulated knowledge Intrinsic motivation | Self-efficacy is inversely associated with school dropout.  Students with high self-efficacy are less likely to drop out. | Promote well-being and academic performance.   - Accompaniment at different levels. - Improve students' ability to regulate and focus on academic support, new study methods and better self-organization. - Psychological counseling, to manage emotions and stressors related to college. |
| Cădariu and Rad, 2023 | Romania  154 | Not defined | Person's belief in his or her ability to achieve goals and complete tasks | Academic | Transversal | Mediator between satisfaction and dropout | Satisfaction  Perceived family acceptance | Academic self-efficacy correlates positively and significantly with satisfaction and with the intention to persist (students with higher self-efficacy tend to be more satisfied and to have a higher intention to continue in their studies).  Academic self-efficacy fully mediates this relationship, highlighting that confidence in one's own abilities is key to academic adjustment and persistence, especially when students are satisfied with their choice of major. | Measures to improve self-efficacy   - Improve students' confidence in their own abilities to succeed academically. - Provide targeted interventions and support programs to promote academic persistence and success. - Create an environment that fosters student satisfaction and engagement with their chosen field of study (by improving the quality of teaching, curriculum design, and the provision of resources and support services tailored to students' needs and interests). |
| Girelli et al., 2018 | Italy  388 | Not defined | A student's belief in his or her ability to organize and perform a specific task. | Academic | Longitudinal | Mediator | Academic adjustment  Perceived autonomy | Autonomous motivation and self-efficacy were significantly negatively associated with intention to quit.  Students who attended college for autonomous reasons and were more confident in their academic abilities were less likely to develop intentions to drop out. | Interventions to prevent dropout intentions:  Foster autonomous motivation and self-efficacy: Improve autonomy support for students by teachers and family members.  Parent training: Implement programs that help parents recognize their children's feelings, provide choices, encourage them, and explain the rationale behind their requests.  Support autonomy in the educational setting: offer choices about how to carry out activities, provide explanatory reasons, use non-controlling language, allow work at the student's pace, and adapt teaching to their preferences.  Teacher training: Develop training protocols for teachers to promote student autonomy.  Actions at university level: Implement programs to improve students' self-efficacy and academic motivation, promoting early academic adjustment. |
| Gonzalez-Perez et al., 2022 | ---  34 | The decision to drop out of college was a process by which a student experiencing lack of motivation decided not to continue his or her studies. | Self-efficacy is an aspect of social cognitive theory defined as "the exercise of human agency through people's beliefs in their abilities to produce desired effects through their actions" ( Bandura, 1997 ) or "judgments about how well one can execute courses of action required to deal with prospective situations" ( Bandura, 1982 ). | Academic | Transversal | Moderator | Sense of belonging | Low levels of motivation affect the intention to drop out of school. This lack of motivation influences the feeling of belonging and the perception of self-efficacy. | Measures to improve self-efficacy:   - Activities in which success can be recognized (mastery experiences), - Mentoring programs (social modeling), - Teachers focused on student success and ability to perform difficult tasks (verbal persuasion) - Promotion of positive emotions through interventions to foster a growth mindset that emphasizes that intelligence and ability are not fixed traits and positive psychological programs (emotional and physiological states) to positively influence their criteria for judging their ability and vulnerability. |
| Heritage et al., 2023 | Australia  196 | Conceptualized student retention as synonymous with other related terms, such as student withdrawal, dropout, and dropout.  We address retention at the university level, so that a student is retained if they remain at their university to complete their degree. | students' perceptions of their ability to perform tasks necessary for academic success. | Academic | Transversal | Mediator | Adjustment | Self-efficacy is related to the intention to remain in studies and acts as a mediator between adjustment-dropout. | Measures to improve self-efficacy   - Professional advice. - Authentic assessment and provide opportunities for work-integrated learning and internships to better connect course content to students' career aspirations. - Provide realistic previews of courses so students can make informed enrollment decisions. - Offer college orientation weeks. - Encourage student groups and associations |
| Lopez-Aguilar and Alvarez-Perez, 2021 | Spain  475 | Not defined | Following Bandura (1997), academic self-efficacy is defined as the beliefs held by students about  their own ability to perform the academic activities required of them in school contexts.  Weinberg, Gould, Yukelson, and Jackson (1981) have defined self-efficacy as the certainty that a person has about his or her  person to be able to perform certain tasks satisfactorily. | Academic | Transversal | Moderator | Self-efficacy moderates the relationship between commitment and performance  And performance is related to the intention to quit  Self-efficacy moderates the relationship between well-being and self-regulation  Self-efficacy moderates the relationship between coping and dropout | Loss of self-efficacy negatively impacts students' cognitive skills and responsibility towards their learning tasks. Those with low self-efficacy and motivation face difficulties in their formative process, which may lead them to consider dropping out. Perceived self-efficacy directly influences academic performance expectations.  Students with high self-efficacy show greater engagement and respond satisfactorily to academic demands.  Learner well-being is related to their self-regulation of learning and self-efficacy. A proactive coping attitude and effective engagement are key to dealing with challenges and emotional stress. | To improve self-efficacy   - Advise students to learn to manage and self-regulate their learning process, and learn to control the impact of high levels of emotional exhaustion and academic burnout. |
| López-Angulo et al., 2023. | Chile  956 | the intention to drop out can be considered as a general construct that accounts for intentions to leave the university definitively  Intent to drop out refers to the thoughts, desires and intentions experienced by students in college regarding the possibility of withdrawing from their degree program before graduation, or leaving a higher education institution  The intention to drop out is also understood as part of a decision-making process that develops in the early stages of the college experience and is characteristically dynamic and convergent with multiple factors | Beliefs, perceptions or expectations that individuals have about their capacity to organize actions and efforts required to materialize a specific type of achievement ( Bandura, 1997 ). This implies having the ability, self-confidence and perceived capacity to grasp and predict social situations, expressed through behavior that is demonstrative of the individual's adaptation to social situations ( Bandura, 1997 ). | Social interactions | Transversal | RD | There is a direct relationship between self-efficacy and intention to quit.  Social satisfaction mediated relationships between social self-efficacy and academic purposes with intention to drop out of school | Social self-efficacy has significant indirect effects on career satisfaction and intention to drop out, mediated by university social satisfaction. This highlights the importance of beliefs and confidence in the ability to understand and act upon social situations, which facilitates the establishment of interpersonal relationships in the university environment.  Interactions and experiences in the university community provide students with models and opportunities that generate learned behaviors, helping them feel competent to meet academic demands.  Perceived academic performance mediates the relationship between social self-efficacy, career satisfaction and dropout intention. Despite the relevance of social self-efficacy for social insertion, perceived academic performance does not act as a mediator in this dynamic | To prevent dropout   - Meet social, psychological and educational support needs. - Minimize inequalities in access to digital resources such as the Internet or computers (private-public offers, rural-urban) |
| Marczuk and Strauss, 2023 | Germany  24,000 approx. | Not defined | belief in one's own ability to master prospective (study) situations | Academic | Transversal | Moderator | Self-efficacy moderates the relationship between performance/satisfaction and intention to quit | Good career guidance benefits students in their decision to continue their studies.  High levels of performance demands  Highly structured programs increase self-efficacy and performance and prevent dropouts | To improve self-efficacy   - Gender-specific policy interventions such as tutorial support (to reduce the level of stress and increase their ability to cope) or learning communities - Employ extracurricular services, such as cognitive, behavioral, and mindfulness interventions, to reduce student anxiety. |
| Morelli et al., 2021 | Italy  431 | Dropout is not a single process, but the final phase of a dynamic, cumulative and multifactorial process of student disengagement. | Self-efficacy for self-regulated learning assesses students' beliefs in structuring environments conducive to learning and in planning and organizing their academic activities. | Self-regulation | Transversal | Mediator and moderator between locus of control, engagement, academic interaction and satisfaction. | Influence of perceived autonomy support, self-efficacy and academic satisfaction on the intention of university students to remain in college. | The intention to drop out is negatively correlated with self-efficacy. Students who feel more self-efficacious are less likely to intend to drop out of college. | To improve self-efficacy   - Inter-course peer tutoring. - Precise and explicit indications to teachers about the objectives of the subject and the study material and guidelines on how to provide periodic feedback on study and learning strategies.   To prevent dropout   - Promote academic integration and social relations among students. - Interventions to improve students' institutional commitment. - Modify students' style of causal attribution of their academic failure. - Develop and implement effective preventive programs and interventions aimed at reducing academic failure and promoting academic wellbeing. |
| Morelli et al., 2023 | Italy  404 | The intention to leave the university does not consist of a single event, but is the final phase of a dynamic, cumulative and multifactorial process of student disengagement. | reence that one can effectively organize and execute a series of actions to adequately face new situations, tests and challenges. | Self-regulation | Transversal | Direct relationship moderated by social relationships | Social relationships (having friends in college) moderate the relationship between self-efficacy and intention to drop out.  Having friends in college may exert a protective effect on the relationship between self-efficacy for self-regulated learning and intention to drop out of college | Intention to drop out of college was positively correlated with age, demotivation, and external motivation, and negatively correlated with self-regulated learning self-efficacy, identified motivation, intrinsic motivation, and college friendships.  Having a high number of friends protects students with low self-efficacy from dropping out of school, while having few friends increases the risk of dropping out. | The same as Morelli et al., 2021. |
| Nemtcan et al., 2020 | Norwey  756 | Dropping out can be defined as leaving an academic institution before completing a degree, with no concrete intentions of returning to higher education.  Transfer refers to the change from one university (where students began their studies) to another institution of higher education. | The concept of self-efficacy refers to the conviction or belief that one can successfully perform a behavior required to achieve a desired outcome (Bandura, 1977). Extensive research evidence indicates a crucial role of self-efficacy in human agency, including behavioral choice, effort, persistence, commitment, and emotional reactions. | Academic | Transversal | Mediator | Time management | Time management skills are positively related to academic self-efficacy, which in turn was negatively related to dropout intentions | There is no |
| Nemtcan et al., 2022  Procastination | Norwey  693 | It distinguishes between two main categories of student exit, institutional exit and exit from the system . The first type of exit describes a pattern of attrition when students change academic institutions (i.e., transfer), while the second distinguishes students who leave the overall educational system altogether (i.e., drop out of college). | An individual's confidence in the ability to solve a problem or achieve a desired goal. | Academic | Transversal | Mediator | Procastination | Academic self-efficacy is negatively related to procrastination and dropout intentions. | To prevent dropout   - Academic tutoring programs focused on academic skills |
| Óturai et al., 2023 | Norwey  264 | Not defined | Belief in one's own ability to perform a specific behavior | Academic | Transversal | Direct relationship | General qualifications  Mathematical self-efficacy | Students' overall efficacy was related to their intentions to drop out. | To improve self-efficacy   - Fostering mathematical self-efficacy in high school students. |
| Van Herpen et al., 2017 | Netherlands  453 | Not defined | Academic self-efficacy, or students' perception of their ability to learn and perform, is another important non-cognitive factor in predicting academic success  Students' perceptions of their level of self-efficacy are based on their past performance, the performance of others, feedback from others about their abilities and performances, and their own feelings about tasks or performance. | Academic | Transversal | No relationship | Effort  Self-efficacy  Previous academic achievement | Pre-college effort and academic self-efficacy prior to matriculation were positively correlated with academic success in the first year. This suggests that greater effort and self-efficacy are associated with better performance.  No statistically significant differences were found in academic success related to self-efficacy, indicating that the level of self-efficacy when applying to college did not influence the probability of dropping out or failing. | They are not proposed |
| Vidal et al., 2022 | Ecuador  629 | Not defined | Not defined | Academic | Transversal | RD | Autoregulation | Students who dropped out scored significantly lower on learning self-regulation strategies, intrinsic value, and self-efficacy.  The vulnerable population had the lowest self-efficacy scores, so vulnerability and social exclusion are academic risk factors for new college students | To prevent dropout   - Measures to support disadvantaged groups of students through motivation and self-regulated learning, emotional intelligence empowerment and the impact of adaptive academic attributions. |
